# 

**Published:** 1901-01-26

**Authors:** 


### NOTICE TO CORRESPONDENTS.

All MSS., Letters, Books for Review, and other matters intended for the Editor should be addressed THE EDITOR, "THE HOSPITAL," THE HOSPITAL BUILDING, 28 & 29 SOUTHAMPTON STREET, STRAND, W.C.

The Editor cannot undertake to return rejected MS., even when accompanied by stamped directed envelope.

**Notice to Advertisers and Subscribers.**

All Advertisements, Orders for Copies of THE HOSPITAL, and Business Communications should be addressed to **THE MANAGER (Not to the Editor),** THE HOSPITAL, 28 & 29 Southampton Street Strand, London, W.C.

The Cost of Subscription, post free, is as follows:—Per week, 2½d.; per quarter, 2s. 8d.; per half-year, 5s. 3d.; per annum, 10s. 6d. Foreign:—Per annum, 15s. 2d., payable, in all cases, in advance.

**NOTICE.—Vol. XXVIII. of "The Hospital" (April to September, 1900), in handsome cloth binding, is now on sale at the Office of this Journal, price, 7s. 6d. Cases for binding the Numbers contained in this Volume 1s. 6d. Index to Vol. XXVIII., 2½d., post free.**

**The Monthly Part for February will be ready on February 1st. Price 6d., by post 8d.**

### NOTES AND NEWS.

HOSPITAL MEETINGS.

### BOOKS RECEIVED.

THE TRUSTEES OF THE BOSTON CITY HOSPITAL.

"Medical and Surgical Reports." Edited by Herbert L. Burrell, M.D., W. T. Councilman, M.D., and Charles F. Wittington, M.D.

LONGMANS, GREEN AND CO.

"Diseases of the Anus and Rectum." By D. H. Goodsall, F.R.C.S., and W. Ernest Miles, F.R.C.S. In two parts. Part I.

BAIRD, LIMITED.

"Meat Inspection: A Report." By Henry O'Neill, M.D.

T. B. BROWNE.

"The Advertisers' A B C."

ADAM AND CHARLES BLACK.

"On Sanitary and Other Matters." By George S. Keith, M.D., LL.D., F.R.C.P.E.

H. K. LEWIS.

"Diseases of the Thyroid Gland." Part I., "Myxœdema and Cretinism." By George R. Murray, M.A., M.D. Camb., F.R.C.P.

### The Student of Medicine.

### APPOINTMENTS.

**Bernard E. G. Bailey,** M.R.C.S., L.R.C.P., appointed House Physician to the East London Hospital for Children, Shadwell. **Clarence Beesley,** L.R.C.P., L.R.C.S., D.P.H.Camb., appointed Medical Officer of Health to the Budleigh Salterton Urban District. **William Moss Bristow,** appointed Honorary Medical Officer to the Victoria Central Hospital, Liscard. **Frederick Constant,** L.D.S.Eng., appointed Honorary Dental Surgeon to the Gravesend Hospital. **G. O. M. Dickenson,** M.B., B.S.Durh., appointed Assistant Medical Officer to the Northumberland County Asylum, Morpeth. **J. G. S. Forrest,** L.R.C.P.Lond., M.R.C.S.Eng., appointed Medical Officer to the Thingoe Union Workhouse. **A. H. Frere,** M.B., C.M.Edin., appointed Certifying Factory Surgeon for the Diss District, Norfolk. **T. W. Heywood,** L.R.C.P.I., M.R.C.S.Eng., appointed Certifying Factory Surgeon for the Borough of Darwen. **T. Gordon Kelly,** B.A., M.D., appointed Medical Officer of Health to the Market Bosworth Rural District Council. **H. E. Gough,** L.R.C.P.Lond., M.R.C.S.Eng., appointed Medical Officer and Public Vaccinator for the Northwich District of the Northwich Union. **H. A. Lane,** M.R.C.S., L.R.C.P.Lond., D.P.H.Camb., appointed Medical Officer and Public Vaccinator for the Western District of the Mile End Union. **Thomas David Lister,** M.D.Lond., M.R.C.P.Lond., appointed Assistant Physician to the Royal Hospital for Children and Women, Waterloo Road.

